# Hematomyelia associated with coronavirus disease 2019: A rare case report

**DOI:** 10.1097/MD.0000000000034197

**Published:** 2023-07-07

**Authors:** Lin-Ming Zhang, Huan-Bo Zhang, Fu-Rong Fan, Ming-Wei Liu

**Affiliations:** a Department of Neurology, the First Affiliated Hospital of Kunming Medical University, Kunming, China; b Trauma Center, the First Affiliated Hospital of Kunming Medical University, Kunming, China; c Department of Emergency Medicine, the First Affiliated Hospital of Kunming Medical University, Kunming, China.

**Keywords:** case report, coronavirus disease 2019, hematomyelia, pulmonary infection, spinal cord injury

## Abstract

Coronavirus disease 2019 (COVID-19) can damage the central nervous system. Although there have been reports of cerebral hemorrhage and infarction caused by COVID-19, hematomyelia due to COVID-19 has never been reported.

**Patient concerns:**

A 40-year-old male was admitted to the hospital with positive nucleic acid detection for COVID-19 after experiencing fever for 2 weeks, urinary retention, fecal retention, and pain in both lower extremities for a week.

**Diagnoses:**

The patient diagnosis was established using thoracic and lumbar magnetic resonance imaging (MRI). Contrast-enhanced thoracic and lumbar MRI revealed subdural (dorsal predominant) short T1 and slightly long T2 bands in the T12-S2 infundibular canal in the scan field, and the subdural hematoma was yet to be distinguished from other diseases. Spinal cord edema was observed in the left vertebral plate and facet joint of the T11 vertebral body, indicative of inflammation. The cerebrospinal fluid (CSF) was positive for COVID-19 nucleic acid.

**Interventions:**

Antiinfection, immunomodulation, correction of acid-base balance and electrolyte disorders, improvement of circulation, nerve nutrition, and other symptomatic supportive treatments were administered to the patient.

**Outcomes:**

The patient symptoms significantly improved after 4 weeks of anti-infection and immunomodulatory therapy. Repeat thoracolumbar MRI revealed absorption of the spinal cord hematoma, and the patient was discharged from the hospital. To date, COVID-19-related hematomyelia has not been reported and anti-infective and immunomodulatory therapies may be effective.

**Lessons:**

COVID-19 not only easily leads to brain injury but can also cause spinal cord injury and even spinal cord hemorrhage. When patients with COVID-19 experience symptoms and signs of spinal cord injury, spinal cord injury and bleeding caused by COVID-19 should be considered, and MRI and lumbar puncture should be performed as soon as possible to make a clear diagnosis.

## 1. Introduction

Coronavirus disease 2019 (COVID-19), caused by severe acute respiratory syndrome coronavirus 2 (SARS-CoV-2), has resulted in a widespread outbreak. Although COVID-19 mainly presents with respiratory symptoms, the target angiotensin-converting enzyme 2 (ACE2) receptor of SARS-COV-2 is expressed in multiple systems throughout the body and can damage various organs, such as the liver, spleen, kidney, digestive tract, heart, hematopoietic system, and brain.^[1]^ Related studies have reported that approximately 36.4% of patients with COVID-19 develop neurological symptoms, such as headache, dizziness, impaired consciousness, acute cerebrovascular disease, ataxia, epilepsy, and neuromuscular damage.^[2]^ Currently, direct and indirect damage are considered to be potential pathogenic mechanisms. Direct injury might occur due to direct invasion of the nervous system by SARS-CoV-2, while indirect injury might be associated with an inflammatory factor storm, hypoxemia, and abnormal coagulation.

Ischemic stroke, peripheral neuroinflammation, hemorrhagic stroke, encephalitis, and Guillain-Barrés syndrome have been reported in cases of COVID-19 accompanied by neurological injury. To date, COVID-19 with hematomyelia have been reported.

## 2. Case report

### 2.1. Ethics approval and consent to participate

Informed written consent was obtained from the patient for the publication of this case report and the accompanying images. This study was reviewed and approved by the local ethics committee of the First Affiliated Hospital of Kunming Medical University. The procedures were in accordance with the Helsinki Declaration of 1975, as revised in 2000.

### 2.2. Case history

A 40-year-old male patient developed a fever 2 weeks prior after catching a cold, with a maximum temperature of approximately 38.4°C. The fever was accompanied by dizziness and fatigue. The patient did not visit a doctor because the fever lasted only a day. The patient developed fever again on December 1, 2022, with a maximum temperature of 37.5°C. The patient faced difficulty urinating and passing stools in the morning. Only a small amount of urine was excreted after uresis under extra energization, and the patient experienced pin-prick-like radiating pain from the lumbosacral area to bilateral thighs, thereby affecting his daily activities. However, the patient did not experience any limb weakness, numbness, or twitching. The patient did not pass stool for a week. The patient took “Bitter Herb Tablets, Herba Lysimachiae Granules”; however, the symptoms did not resolve as urinary retention progressed. On December 18, 2022, the patient visited the Department of Neurology at the First Affiliated Hospital of Kunming Medical University for treatment.

### 2.3. Past medical history

The patient had hypertension for 3 years, with a maximum blood pressure of 230/160 mm Hg, amlodipine mesylate 10 mg once a day, and recent fluctuations in his blood pressure ranging between 30 and 200/90 and 110 mm Hg. The patient did not report any history of trauma, surgery, blood transfusion, or allergy, and the history of vaccination was unknown.

### 2.4. Physical examination

The patient was conscious, had poor mental health, had a Glasgow Coma Scale score of 15, and was fluent in speech and cooperative during physical examination. Physical examination revealed a normal orientation, calculation ability, and memory. The neck was soft without resistance, with negative Kernig and Brudzidski signs. The bilateral pupils were equal, round, and reactive to light (d = 3 mm); direct and indirect light reflexes were present, and the patient could perform bilateral visual activities. Diplopia and nystagmus were not observed, visual field and vision were normal under rough access, bilateral forehead wrinkles were symmetrical, bilateral eye closure was forceful, bilateral nasolabial sulcus was symmetrical, and the angle of the mouth was not deflected. Additionally, no leakage of air was observed after cheek blowing, the chewing was forceful, the tongue was centered, the bilateral soft palate elevation was normal, the uvula was centered, and bilateral pharyngeal reflex was observed (++), along with strong head and shoulder elevation and neck rotation. The limbs and trunk muscles did not show signs of atrophy; grade 5 muscle strength was observed in the distal proximal part of the bilateral upper limbs, the patient experienced pain in the proximal part of the bilateral lower limbs, the distal muscle strength was normal, the muscle tone was normal, tendon reflex was observed (++), and the pathological sign was negative. The finger-nose test was stable and accurate, the muscle retraction test revealed a negative result, the alternation test was bilaterally symmetrical without clumsiness, the heel-knee-shin test was stable, the patient was stable while standing with his eyes open and closed, with a normal gait, sensation of pain and temperature, and the touch pressure sensation of the limbs was normal. The pressure, position, and vibration thresholds were acceptable in the rough test, and scratches on the skin were not abnormal. The patient had a grade 1 score for the water swallow test, a modified Rankin scale score of 4, and no significant anxiety or depression.

### 2.5. Laboratory data

On January 18, 2023, laboratory test results were as follows: White blood cell count, 9.79 × 10^9^/L (neutrophils, 78.3%; lymphocytes, 9.9%); red blood cell count, 6.43 × 10^12^/L; hemoglobin, 190 g/L; platelet count, 349 × 10^9^/L; procalcitonin level, <0.05 mg/L; C-reactive protein, 26.5 pg/L. The coagulation and fibrinolytic system test results were as follows: fibrin degradation product level, 1.4 mg/L; D-dimer, 0.49 mg/L; antithrombin III, 85%; prothrombin time, 11.7 seconds; prothrombin activity, 126%; international standard ratio, 0.89; prothrombin time ratio, 0.91; international sensitivity index, 1.29; fibrinogen, 4.29 g/L; clotting time, 19.7 seconds; activated partial thromboplastin time, 45.1 second. Liver and kidney function test results, electrolyte levels, and cardiac enzyme results were normal. On January 20, 2023, routine cerebrospinal fluid (CSF) test results were as follows: turbid and red fluid with positive Pan test (+); White blood cell count, 1038 × 10^6^/L (neutrophils, 50%; large lymphocytes, 19%); red blood cell count, 299063 × 10^6^/L; *Cryptococcus* was not detected on CSF ink staining, and no fungal spores or mycelia were detected on CSF analysis. The CSF biochemistry results were as follows: glucose, 3.72 mmol/L; chloride ions, 84.70 mmol/L; total microprotein, 3.200 g/L; microalbumin, 1834.9 mg/L; adenosine deaminase, 7.3 U/L; lactate dehydrogenase, 1208 U/L; aspartate aminotransferase, 59.7 U/L; alanine aminotransferase, 6.2 U/L; aspartate aminotransferase/alanine aminotransferase, 9.63; phosphocreatine kinase, 73.8 U/L. Antibodies associated with tuberculosis, systemic lupus erythematosus, rheumatoid arthritis, anticardiolipin, and antineutrophil cytoplasmic antibodies were not detected. The results of the new COVID-19 nucleic acid test were positive.

### 2.6. Imaging data

**Figure 1.**
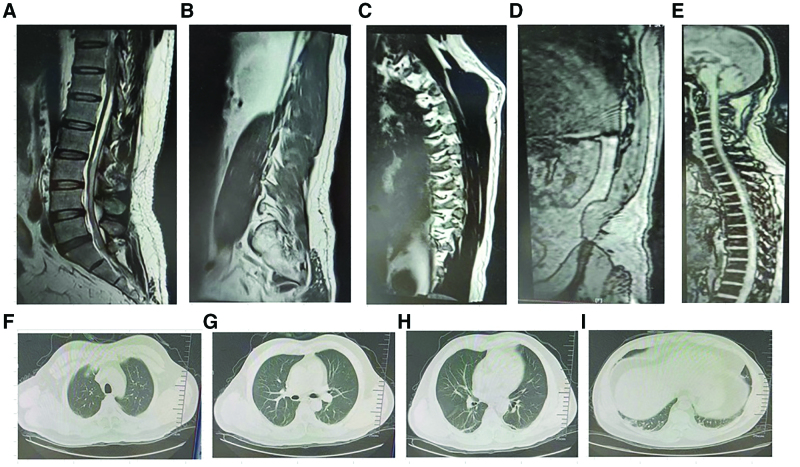
Changes in the chest computed tomography (CT) and magnetic resonance imaging (MRI) of the thoracic and lumbar spine at the time of patient admission. (A–E) Changes in the thoracic and lumbar spine MRI at the time of patient admission. (F–I) Changes in chest CT of the patient upon admission.

The abdominal ultrasound findings were as follows: increased bilateral kidney volumes (combined with renal function), patchy strong echogenicity in the left kidney (small stones observed), and extremely full bladder (possible urinary retention). The thoracic and lumbar contrast-enhanced and conventional magnetic resonance imaging (MRI) results were as follows: a nodular and slightly low-signal shadow was observed in the left part of the spinal canal at the T11 level (vertical diameter × transverse diameter × anteroposterior diameter, 1.2 × 0.6 × 0.5 cm). Contrast-enhanced MRI revealed marginal enhancement and high-signal T2 bands in the medullary cone in the same layers. Contrast-enhanced MRI did not reveal any obvious enhancement. The scanning field was set within the thoracic and lumbar spinal membranes and part of the end-filament of the cauda equina. These findings are indicative of inflammation. Subdural (dorsally predominant) short T1 and slightly long T2 bands were observed in the T12-S2 infundibular canal in the scan field, and the subdural hematoma was yet to be distinguished from other diseases. Spinal cord edema was observed in the left vertebral plate and facet joint of the T11 vertebral body, indicative of inflammation (Fig. 1A–E). Chest computed tomography (CT) revealed subpleural lesions in the lower lobe of the bilateral lungs and the upper lobe of the left lung (Fig. 1F–I).

### 2.7. Diagnosis and treatment

**Figure 2.**
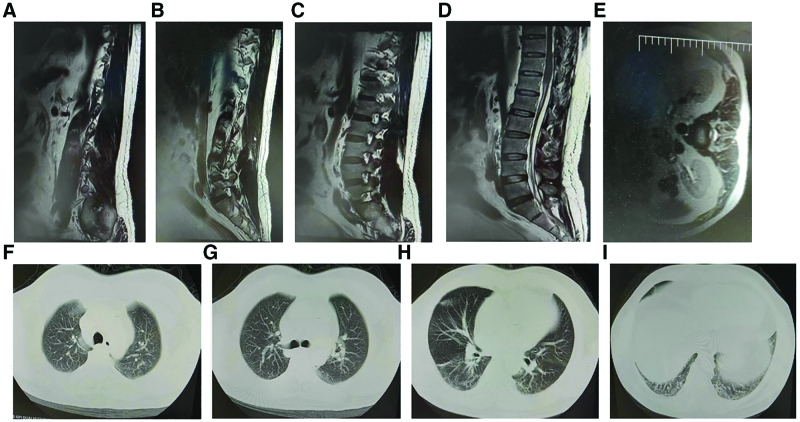
Changes in the chest computed tomography (CT) and magnetic resonance imaging (MRI) of the thoracic and lumbar spine after 1 wk of treatment. (A–E) Changes in the thoracic and lumbar spine MRI after 1 wk of treatment. (F–I) Changes in chest CT after 1 wk of treatment.

**Figure 3.**
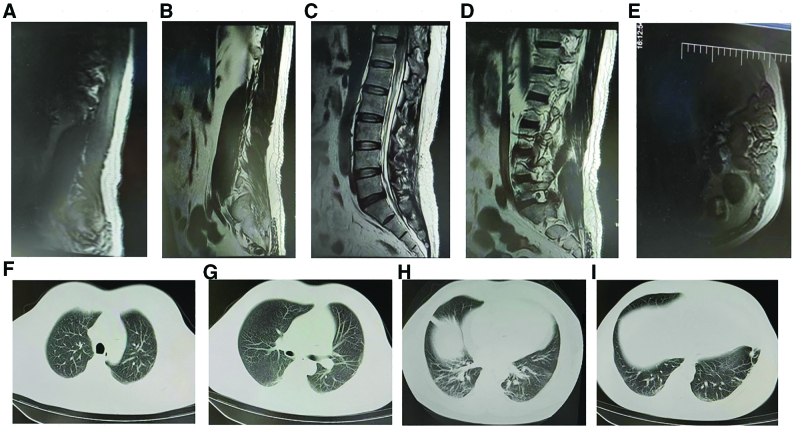
Changes in chest computed tomography (CT) and magnetic resonance imaging (MRI) of the thoracic and lumbar spine after 3 wk of treatment. (A–E) Changes in the thoracic and lumbar spine MRI after 3 wk of treatment. (F–I) Changes in the chest CT after 3 wk of treatment.

According to the above information, the patient was initially diagnosed with the following conditions:

Acute hematomyelia, T11 spinal cord medullary edema, severe pulmonary infection, and COVID-19. Ceftriaxone was administered to the patient after admission, which was then adjusted to cefoperazone sodium/sulbactam sodium to treat the infection, provide nerve nutrition, improve neurological microcirculation, and correct the electrolyte and acid-base balance. After 2 weeks of treatment, the thoracolumbar spine MRI was reviewed, which revealed a decrease in the T11 spinal cord edema, with absorption of the subdural hematoma in the T12-S2 infundibular canal compared with the absorption observed in the hospital (Fig. 2A–E). The COVID-19 nucleic acid test results were negative. The lung exudate increased and the infection worsened (Fig. 2F–I). noninvasive ventilator-assisted breathing was provided, meropenem was administered for infection control, nerve nutrition was provided, and improvement was observed in the neurological microcirculation. reexamination of chest CT after 2 weeks of treatment revealed significant resolution of the pulmonary infection (Fig. 3A–E), Thoracolumbar MRI still revealed spinal cord edema at the level of T11, and complete absorption of the subdural hematoma was observed in the T12-S2 subdural duct (Fig. 3F–I). The patient was weaned off the ventilator, and dyspnea improved significantly; therefore, the patient was discharged.

### 2.8. Post-treatment follow-up

Complete absorption of the spinal canal subdural hematoma at the level of T12-S2 was observed on thoracolumbar MRI after 4 weeks of treatment. The patient did not report any discomfort during the 3-month follow-up.

## 3. Discussion

COVID-19 is mostly associated with respiratory symptoms, and SARS-CoV-2 can be detected in the bronchoalveolar lavage, sputum, saliva, and nasopharyngeal swabs of patients. COVID-19 primarily affects the lungs, and although most patients present with mild clinical symptoms, some cases progress to pneumonia, acute respiratory distress syndrome, and even death.^[3]^ As the epidemic is widespread, several clinical studies have reported that patients with SARS-Co V-2 infection experience acute injury to extra-pulmonary organs, including the heart, gastrointestinal tract, liver, kidneys, and nervous system.^[4,5]^ Among patients with an injured nervous system, brain injury and neuromuscular disease are common,^[6]^ and COVID-19-associated spinal cord injury and hemorrhage have not been reported to date.

Increasing clinical evidence has reported that SARS-CoV-2 can invade the nervous system, and a retrospective study of 214 patients with COVID-19 reported 78 (36.4%) cases with neurological involvement, of which 28.2% had severe central nervous system injury.^[7]^ Studart-Neto et al retrospectively analyzed 1208 patients with COVID-19 and reported 89 cases (7.4%) that presented with neurological manifestations, including encephalopathy (44.4%), stroke (16.7%), epilepsy (9.0%), and neuromuscular disease (5.6%).^[8]^ These neurological manifestations frequently occur in elderly patients and individuals with multiple co-morbidities or severe infections, with headache, olfactory deficits, and myalgia being the most common manifestations. Takeshi et al detected SARS-CoV-2 ribonucleic acid in CSF specimens, providing direct evidence of SARS-CoV-2 neuroinvasive nature.^[9]^ In another study, a polymerase chain reaction was performed on CSF specimens of 303 patients, of which 6% of the patients tested positive for SARS-CoV-2, and all patients exhibited central nervous system localization. Antibody testing of CSF was performed in 58 patients, and 72% tested positive for SARS-CoV-2 antibodies.^[10]^ Egbert et al observed abnormal brain signals in 124 of 361 patients who underwent COVID-19 neuroimaging studies, with the most common abnormalities being white matter hyperintensities on MRI (53%) and hypointensities on CT scans (24%). The white matter abnormalities were commonly observed in the anterior and posterior white matter of the bilateral brain, along with microhemorrhages, hemorrhages, and infarcts.^[11]^ Matschke et al found SARS-CoV-2 ribonucleic acid in 53% of autopsied brains.^[12]^ The K18h ACE2 transgenic mouse model was used to demonstrate that SARS-CoV-2 infects the brain in addition to the lung.^[13]^ All the above clinical evidence proves that SARS-CoV-2 can invade the human nervous system and cause neurological impairment. However, no COVID-19-related spinal cord injury or hemorrhage has been reported in studies conducted in our country or internationally. Herein, we report for the first time that COVID-19 causes spinal cord injury and hemorrhage.

It is still believed that angiotensin converting enzyme-2 receptor binding is a key factor in SARS-CoV-2 viral infectivity and multiorgan damage, and the structure and receptor binding domain of SARS-CoV-2 are similar to those of SARS-CoV in that spinosin transduces the virus into the nervous system by binding to the ACE2 receptor of the host cell after breaching the host anatomical and chemical defense barriers.^[14,15]^ No specific medication treats COVID-19. Herein, anti-inflammatory, immunomodulatory, nerve nutrition, and symptomatic supportive therapies were administered to the patient, along with efforts to improve neurological microcirculation; however, the pulmonary infection worsened, following which anti-infection and anticoagulation therapies were administered to the patient along with ventilation provided in the prone position. The pulmonary lesions showed significant absorption, with resolution of hematomyelia and edema, and the patient was discharged after significant improvement in symptoms.

In summary, hematomyelia due to COVID-19 has not been reported, and anti-infective and immunomodulatory therapies may be effective. However, further confirmation with a larger sample size and further validation in animal studies and clinical trials are required. This study presents and analyzes the etiology, pathogenesis, and treatment of patients with COVID-19-associated hematomyelia to improve our understanding of the developmental mechanisms, treatment, and prognosis of these patients.

## Acknowledgments

This work was supported by Nature Science Foundation of China under Grant No. 81960350 and Grant No. 82060252; the Yunnan Applied Basic Research Project - Union Foundation of China under Grant No. 202201AY070001-091; Major Science and Technology Special Project of Yunnan Province (Grant No. 202102AA100061); Yunnan Basic Research Projects (Grant No. 2018FB115); the Yunnan health training project of high-level talents (Grant No. H-2018058); and the Applied Basic Research of Yunnan Neurological Disease Diagnosis and Treatment Center (Grant No. ZX2019-03-05).

## Author contributions

**Conceptualization:** Lin-Ming Zhang, Huan-Bo Zhang, Ming-Wei Liu.

**Formal analysis:** Lin-Ming Zhang, Huan-Bo Zhang, Ming-Wei Liu.

**Investigation:** Lin-Ming Zhang, Fu-Rong Fan, Ming-Wei Liu.

**Methodology:** Huan-Bo Zhang, Fu-Rong Fan.

**Project administration:** Huan-Bo Zhang.

**Resources:** Huan-Bo Zhang, Fu-Rong Fan, Ming-Wei Liu.

**Software:** Lin-Ming Zhang, Huan-Bo Zhang.

**Supervision:** Ming-Wei Liu.

**Validation:** Huan-Bo Zhang, Fu-Rong Fan.

**Visualization:** Lin-Ming Zhang, Huan-Bo Zhang, Fu-Rong Fan.

**Writing – original draft:** Fu-Rong Fan, Ming-Wei Liu.

**Writing – review & editing:** Ming-Wei Liu.
